# Development and Implementation of the Selection, Entry, Lithotripsy, Exit, and CT (SELECT) Mnemonic and Checklist: A Structured Approach to Urolithiasis Assessment and Management for Urology Trainees and Junior Urologists

**DOI:** 10.7759/cureus.101200

**Published:** 2026-01-09

**Authors:** Abdullatif E Al-Terki, Said Yaiesh, Rehan N Khan, Sharifah Maqames, Tariq F Al-Shaiji, Naser Al-Soudan Al-Anzi

**Affiliations:** 1 Urology Unit, Department of Surgery, Amiri Hospital, Kuwait City, KWT; 2 Urology Unit, Department of Surgery, Jaber Al Ahmad Hospital, Kuwait City, KWT; 3 Kuwait Board of Urology, Kuwait Institute for Medical Specialization, Kuwait City, KWT

**Keywords:** endourology, mnemonic, surgical education, urolithiasis, urology

## Abstract

Introduction

Urolithiasis is a common condition in urological practice, with a particularly high burden in the Middle East due to dietary and lifestyle factors. Effective evaluation and management require a systematic approach, yet trainees often struggle with decision-making due to patient- and stone-related variability. To address these challenges, we developed the selection, entry, lithotripsy, exit, and CT (SELECT) mnemonic and checklist, a structured five-tier systematic approach designed to streamline renal stone assessment and management.

Methods

A panel of endourologists and educators identified key challenges in urolithiasis management among urology residents. A pre-implementation survey was conducted among 28 residents in the Kuwait Board of Urology program to assess confidence and challenges in renal stone evaluation and keenness for a mnemonic. Following the development of the SELECT mnemonic and checklist, trainees were introduced to the tool and instructed to utilize it over four weeks. A post-implementation survey was then conducted, and feedback was collected from both trainees and training supervisors.

Results

Before implementation, only 25% of residents reported confidence in assessing renal stone characteristics and selecting treatment modalities. Post-implementation, 84% reported improved confidence in assessment, and 80% felt more assured in treatment selection. Supervisors also noted improved diagnostic accuracy and decision-making among trainees. The mnemonic reduced reliance on senior guidance, although time constraints were a reported limitation.

Conclusion

The SELECT mnemonic and checklist offer a structured, evidence-based approach to renal stone management, improving trainee confidence and decision-making. External validation and potential scoring integration could further enhance their utility in urological education and clinical practice.

## Introduction

The management of urinary stone disease is the bread and butter of urology practice. In the Middle Eastern region in particular, the burden of urolithiasis is great, partly attributed to dietary and lifestyle factors [1]. The evaluation and management of urolithiasis require a systematic and evidence-based approach to ensure optimal patient outcomes while minimizing interventions [2].

International urological societies, including the American Urological Association (AUA) and the European Association of Urology (EAU), have established best-practice guidelines for the evaluation and management of urolithiasis, yet many trainees and young and early-career professionals face challenges in the assessment of renal stones, selection of the appropriate intervention modality, and planning of follow-up [2,3]. The complexity of such processes in decision-making arises from patient- and stone-related factors, including body habitus, comorbidities, and stone properties such as size, location, composition, and anatomical variations, as well as stone-related surgical history. This introduces a constant variability that impacts prompt management decisions that could eventually delay appropriate treatment [4,5].

To address this gap, we developed the selection, entry, lithotripsy, exit, and CT (SELECT) mnemonic, a five-tier checklist and clinical tool designed to streamline the assessment and management of urolithiasis. An extended checklist of the SELECT mnemonic was also devised. Checklists and mnemonics have been universally shown to improve surgical care and practices due to their ease of implementation and even memorization [6-8]. By integrating evidence-based principles of urolithiasis evaluation and management into a simple, easy-to-remember format, SELECT facilitates efficient and consistent decision-making in both elective and emergency settings.

Here, we present our successful development and implementation of the SELECT mnemonic checklist and its impact on resident confidence and the appropriateness of urolithiasis management.

## Materials and methods

A panel of endourologists and educators led by the authors was assembled by invitation, and a focus group was prompted about identifiable gaps and areas of improvement necessary in the educational process for urology residents with regard to urolithiasis evaluation and management. This was followed by a survey administered to all urology residents in the Kuwait Board of Urology program, aiming to provide valuable insights into the needs of our urology residents and guide the development of a practical tool to enhance confidence and efficiency in renal stone management. The results from the survey were then analyzed, along with the recommendations of the panel, and the SELECT mnemonic and checklist were developed (Tables 1, 2).

**Table 1:** SELECT Mnemonic URS, ureterorenoscopy; RGP, retrograde pyelogram; RIRS, retrograde intrarenal surgery; NCCT-KUB, non-contrast CT of the kidney, ureter, and bladder

SELECT	
Selection	Anatomy: normal, malrotated, horseshoe, and other abnormalities
	Stone: size, location, density, and calyces involved
	Patient history: fitness, prior attempts for the same stone presentation, complications, stent symptoms, and other procedures
Entry	Prior stenting, ureteric orifice assessment (cystoscopic if any, H1-H3), retrograde assessment (if any, including guidewire introduction, semirigid URS, RGP, and access sheath introduction), feasibility for RIRS versus need for percutaneous access, and duality
Lithotripsy	Flexible ureteroscope type or perc size, need for access sheath, irrigation mode, stone displacement (possibility), and laser type/energy/mode
Exit	Clearance (stone-free), negative pressure effects (flushing via ureteric and pulling out), access sheath effects (if any, removal under direct vision), and need for stent
CT	First follow-up: 10 days post-operatively and NCCT-KUB at four weeks post-operatively

**Table 2:** SELECT Checklist An extension of the SELECT mnemonic ASA, American Society of Anesthesiologists; URS, ureterorenoscopy; RGP, retrograde pyelogram

SELECT	
Selection	
Anatomy	Normal/malrotated/horseshoe/others
Stone size	
Stone location	
Stone density (HU)	
Calyces involved	
Patient fitness	ASA I/ASA II/ASA III/ASA IV
Attempt	First/subsequent
Complications (if any)	
Stent-related symptoms	
Alternative to procedure	
Entry	
Prior stenting	Yes/no
Ureteric orifice assessment	H0/H1/H2/H3
Guidewire introduction	Successful/unsuccessful
Semirigid URS ± RGP passive dilatation	
Access sheath introduction	Size (Fr)
Technique	Classical/over semirigid/over flexible
Inspection	
Lithotripsy	
Flexible ureteroscope	Disposable/non-disposable
Scope size	
Irrigation	Gravity/manual/pressure
Stone displacement	Yes/no
Laser modes	Dusting/fragmentation/popcorn
Exit	
Clearance (stone-free)	Flushing/pull access sheath
Need for a stent	Yes/no
CT	
Follow-up (FU)	First FU 10 days/CT plain at four weeks

The urology residents were then introduced to the mnemonic and checklist, and a detailed explanation of each tier was demonstrated in a dedicated discussion during their academic day. From there, the mnemonic and checklist were distributed in both paper and electronic formats for ease of use, and the residents were requested to utilize them at their discretion for a period of four weeks. The residents would use the mnemonic as an on-spot reminder while referring to the checklist should they require to validate more details in their assessments.

At the end of the four-week trial, a post-implementation survey was distributed to all the residents to assess the effectiveness of the SELECT mnemonic and help evaluate and refine this educational tool. An open discussion meeting with all the residents also ensued to receive open-ended feedback and discuss means for improvement. A different post-implementation survey was also distributed to training supervisors in the different training sites for the urology residency program, designed to gather feedback from supervisors on whether the SELECT mnemonic has improved urology residents' ability to assess and manage renal stone cases over the past four weeks. The training supervisors were initially not made aware of the trainees utilizing the mnemonic and checklist to ensure the objective assessment of the trainees' performance. Toward the end of the survey, they were introduced to the mnemonic and checklist and informed that their trainees have been utilizing it recently. Survey responses were analyzed, and descriptive statistics were utilized to present the results.

## Results

A total of 28 urology trainees and residents responded to the initial pre-implementation survey (100% response rate). Over half of the trainees (n = 16) were junior residents in the first two years of training. Trainees answered questions about the frequency and challenges faced during the management of urolithiasis. Eighty-two percent (n = 23) of trainees reported encountering urolithiasis cases daily. Only 25% of trainees reported their absolute confidence in assessing renal stone location, size, and composition or selecting the correct treatment modality on a confidence scale of 1-5, the latter being most confident (n = 7).

When enquired about what aspects of assessment they found most challenging, assessing anatomical factors affecting treatment decisions was the greatest factor of concern (n = 16, 57%), while considering patient comorbidities and preferences and limitations to procedure specifics were aspects that made treatment decisions difficult (n = 12, 43% each). Most trainees reported that they consult a senior physician for advice regarding management plans regularly (n = 21, 75%). An overwhelming majority reported that they would welcome the use of a mnemonic and would find it helpful, particularly if the mnemonic was suitable (n = 25, 89%). Table 3 summarizes data about the respondents to the pre-implementation survey and their responses.

**Table 3:** Responses to Survey of Need Assessment and Pre-implementation of SELECT SELECT, selection, entry, lithotripsy, exit, and CT; PGY, post-graduate year

N = 28	Count (n)	Percentage (%)
Level of training		
PGY1	8	28.6
PGY2	8	28.6
PGY3	5	17.9
PGY4	5	17.9
PGY5/fellow	2	7.0
Questions		
How often do you encounter patients with renal stones in your clinical practice?		
Daily	23	82.1
Weekly	4	14.3
Monthly	1	3.6
Rarely	0	0.0
On a scale of 1-5 (1 = very difficult; 5 = very easy), how confident are you in assessing the size, location, and composition of renal stones?		
1	0	0.0
2	1	3.6
3	6	21.4
4	14	50.0
5	7	25.0
What aspects of renal stone assessment do you find most challenging? (Respondents selected all that apply)		
Understanding image findings	5	17.9
Determining stone composition	13	46.4
Assessing anatomical factors affecting treatment decisions	16	57.1
Predicting stone passage or need for intervention	7	25.0
On a scale of 1-5 (1 = very difficult; 5 = very easy), how confident are you in selecting appropriate treatment modalities for renal stones based on patient and stone characteristics?		
1	0	0.0
2	1	3.6
3	6	21.4
4	14	50.0
5	7	25.0
What factors make deciding on treatment for renal stones most difficult? (Respondents selected all that applied)		
Understanding the indications for each treatment modality	4	14.3
Considering patient comorbidities and preferences	12	42.9
Accounting for stone size, location, and composition	8	28.6
Limited exposure to specific procedures	12	42.9
None of the above/others	7	25.0
How often do you feel you need to consult a senior or attending physician for advice on managing renal stones?		
Always	5	17.9
Often	5	17.9
Sometimes	11	39.2
Rarely	6	21.4
Never	1	3.6
Do you have easy access to senior support or guidelines for renal stone management when needed?		
Always	25	89.3
Yes, but not consistently	3	10.7
No	0	0.0
What resources do you currently use to guide your decisions about renal stone management? (Respondents selected all that applied)		
Senior/attending physician guidance	19	67.9
Clinical guidelines	20	71.4
Textbooks or online resources	13	46.4
Personal experience or judgment	8	28.6
Would a simple mnemonic or checklist for assessing renal stones and determining treatment and follow-up help streamline your clinical decision-making?		
Yes, definitely	15	53.6
Yes, maybe, depends	10	35.7
No	2	7.1

Table 4 summarizes the trainee responses to the post-implementation survey (n = 27; overall response rate of 96%). On a scale of 1-5, 88% reported finding the SELECT mnemonic and checklist helpful, 84% reported that using them improved their confidence in assessing renal stones (score ≥ 3; n = 21), and 80% reported increased confidence in making treatment decisions (score ≥ 3; n = 20). The most common improvement was in selecting the right treatment modality for the encountered stone (n = 11, 41%). The use of the mnemonic and checklist reduced the reliance of the trainees on senior physicians' advice and guidance (n = 16, 62%). No trainee reported difficulty utilizing or memorizing the SELECT mnemonic. However, time constraints were cited by a majority of the trainees in further utilizing the tools (n = 18, 69%). All respondents reported that they would recommend the SELECT mnemonic and checklist to a certain degree to another junior colleague or urology resident.

**Table 4:** Responses to Survey for Post-implementation of SELECT: Residents SELECT: selection, entry, lithotripsy, exit, and CT

N = 27 (unless specifically specified)	Count (n)	Percentage (%)
Questions		
On a scale of 1-5 (1 = not helpful at all; 5 = extremely helpful), how helpful has the SELECT mnemonic been in guiding your renal stone assessment and management decisions? (n = 17)		
1	1	5.9
2	1	5.9
3	8	47.1
4	6	35.3
5	1	5.9
How often have you used the SELECT mnemonic in your clinical practice since its introduction?		
Daily	2	7.4
Weekly	1	3.7
Occasionally	13	48.2
Rarely	4	14.8
Never	7	25.9
Which aspects of renal stone management has SELECT improved for you the most? (Respondents selected all that apply)		
Systematic assessment of stone characteristics	9	33.3
Selecting appropriate treatment modalities	11	40.7
Planning follow-up care	6	22.2
Communication with senior staff and colleagues	6	22.2
Patient counselling	4	14.8
Others	2	7.4
On a scale of 1-5 (1 = not confident; 5 = very confident), how has SELECT affected your confidence in assessing renal stones? (n = 25)		
1	2	8.0
2	2	8.6
3	13	52.0
4	5	20.0
5	3	12.0
On a scale of 1-5 (1 = not confident; 5 = very confident), how has SELECT affected your confidence in deciding on treatment modalities for renal stones? (n = 25)		
1	3	12.0
2	2	8.0
3	10	40.0
4	7	28.0
5	3	12.0
Do you feel that the mnemonic has reduced your reliance on senior guidance or additional resources when managing straightforward renal stone cases? (n = 26)		
Yes, significantly	2	7.7
Yes, to some extent	16	61.5
No, has not made a difference	6	23.1
No, I still rely heavily on senior support	2	7.7
How easy is the SELECT mnemonic to remember and apply in clinical settings? (n = 26)		
Very easy	4	15.4
Somewhat easy	4	15.4
Neutral	18	69.2
Somewhat difficult/difficult	0	0.0
What challenges, if any, have you encountered when applying the SELECT mnemonic in practice? (Respondents selected all that applied)		
Lack of time in a busy clinical setting	18	69.2
Difficulty fitting complex cases into mnemonic/checklist	8	30.8
Others	2	7.7
Lack of time in a busy clinical setting	18	69.2
Would you recommend the SELECT mnemonic to other urology residents or junior colleagues? (n = 26)		
Yes, definitely	10	38.5
Maybe	15	57.7
No	1	3.9

Residents of the Kuwait Board of Urology train in six different clinical sites where they encounter urolithiasis cases in both adult and pediatric patients. All six training supervisors responded to the feedback survey distributed post-implementation, and their responses are summarized in Table 5. Of note, all supervisors reported a level of improvement in the trainees' ability to assess and make management decisions in renal stone cases, as well as demonstrating better accuracy in diagnosis and determining stone characteristics, appropriate decisions regarding treatment modalities, and critical thinking. They also reported a major satisfaction with the SELECT mnemonic outcomes, after they were informed of its recent utilization by the trainees.

**Table 5:** Responses to Survey for Post-implementation of SELECT: Feedback From Training Supervisors SELECT: selection, entry, lithotripsy, exit, and CT

N = 6 (unless specifically specified)	Count (n)	Percentage (%)
Questions		
In the last four weeks, have you noticed an improvement in the residents' ability to assess and manage renal stone cases?		
Yes, significant improvement	3	50.0
Yes, moderate improvement	3	50.0
No noticeable change/decline in performance	0	0.0
In the last four weeks, have the residents demonstrated better accuracy in diagnosing renal stones and determining their characteristics?		
Yes, consistently	6	100.0
Occasionally/no noticeable change	0	0.0
In the last four weeks, have you observed improvements in the residents' decision-making regarding the appropriate modality for renal stone treatment?		
Yes, significant improvement	3	50.0
Yes, moderate improvement	3	50.0
No noticeable change	0	0.0
In the last four weeks, do the residents appear more confident and systematic when independently assessing patients with renal stones?		
Yes	5	83.3
Somewhat	1	16.7
No	0	0.0
In the last four weeks, have you noticed the residents explicitly using a mnemonic or checklist or a systematic approach during discussions or case evaluations?		
Frequently	2	33.3
Occasionally	3	50.0
Rarely	1	16.7
Never	0	0.0
In the last four weeks, has the residents' ability to communicate their assessment and management plans for renal stones to patients improved?		
Yes, significantly	4	66.7
Yes, moderately	2	33.3
No noticeable change	0	0.0
In the last four weeks, has the frequency of residents seeking your input for straightforward renal stone cases decreased?		
Yes, significantly	2	33.3
Yes, somewhat	4	66.7
No noticeable change	0	0.0
They are seeking more input	0	0.0
In the last four weeks, have the residents demonstrated improved critical thinking and clinical reasoning in managing complex renal stone cases?		
Yes, significantly	2	33.3
Yes, somewhat	4	66.7
No noticeable change	0	0.0
Knowing now that the SELECT mnemonic and checklist were introduced, are you satisfied with the overall improvement, if any, in the residents' management of renal stones after the introduction of the SELECT mnemonic?		
Very satisfied	1	16.7
Satisfied	3	50.0
Neutral	2	33.3
Dissatisfied	0	0.0

## Discussion

To our knowledge, this is the first simplified mnemonic and checklist aimed at urology trainees and residents to aid in urolithiasis and renal stone management outside of international guidelines and publications. The justification for developing SELECT stems from the challenges faced by urology residents and trainees in real-world clinical practice. Many junior urologists struggle with interpreting imaging findings, distinguishing between patients requiring active intervention and conservative management, and determining the most appropriate treatment modality based on patient-specific factors. Additionally, access to senior guidance may be inconsistent, particularly in busy training centers or emergency settings where immediate decisions are required [9,10]. Given these realities, there is a critical need for a structured tool that assists trainees in navigating the complexities of renal stone management. SELECT was designed to serve as an educational and clinical aid, promoting systematic evaluation and reducing reliance on subjective decision-making.

The primary aim of this study is to validate the SELECT mnemonic and checklists as structured tools for renal stone assessment and management. The study sought to assess whether their implementation improves the confidence, accuracy, and efficiency of urology trainees in evaluating renal stones and selecting appropriate treatment strategies. The pre- and post-implementation surveys report an overwhelming positive response from the trainees, and that was further validated by the evaluation made by their training supervisors before they were made aware of the utilization of the residents of these new tools. By addressing these objectives, this study establishes the utility of SELECT as a practical clinical tool, enhancing the education and training of urology residents while possibly eventually improving patient care in renal stone management.

Limitations

We are aware of the limitations of these tools' development and of the study. The small number of respondents and the reliance on expert opinion primarily to develop the tool may challenge the reliability of the tools and their assessment. However, these tools do not introduce new clinical concepts but rather appropriately summarize them in a memorable mnemonic and referenceable checklist for the residents. The external validation of the mnemonic and checklist, as well as weighted scoring, would be the next step in solidifying this effort to simplify urolithiasis management learning. Additionally, the findings that trainees face daily difficulties in determining stone composition, best treatment modalities, and probabilities of stone passage indicate that, upon the external validation of SELECT, there is a potential for the transformation or exploration of potential scoring systems to further aid trainees and practitioners in the assessment and management of urolithiasis.

## Conclusions

The SELECT mnemonic and checklists are practical, easy, and impactful tools to aid urology residents and junior professionals in the assessment and management of renal stone disease and urolithiasis. The mnemonic simplifies the patient assessment and decision-making in a five-tier process, with the checklist serving as an extension aid for further details. The respondents and training supervisors reported positive feedback to SELECT, and external validation and scoring algorithms for this tool are the next steps in further incorporating these tools in renal stone urological education and training.
